# Investigation of the Expression of Inflammatory Markers in Oral Biofilm Samples in Patients with Systemic Scleroderma and the Association with Clinical Periodontal Parameters—A Preliminary Study

**DOI:** 10.3390/life11111145

**Published:** 2021-10-27

**Authors:** Mayte Buchbender, Amelie Lugenbühl, Jakob Fehlhofer, Christian Kirschneck, Jutta Ries, Rainer Lutz, Michael Sticherling, Marco Rainer Kesting

**Affiliations:** 1 Department of Oral and Maxillofacial Surgery, University of Erlangen-Nuremberg, 91054 Erlangen, Germany; a.lugenbuehl@googlemail.com (A.L.); jakob.fehlhofer@uk-erlangen.de (J.F.); jutta.ries@uk-erlangen.de (J.R.); Rainer.lutz@uk-erlangen.de (R.L.); marco.kesting@uk-erlangen.de (M.R.K.); 2 Department of Orthodontics, University of Regensburg, 93053 Regensburg, Germany; christian.kirschneck@klinik.uni-regensburg.de; 3 Department of Dermatology, University of Erlangen-Nuremberg, 91054 Erlangen, Germany; michael.sticherling@uk-erlangen.de

**Keywords:** biofilm samples, expression levels of cytokines, periodontal parameters, systemic scleroderma (SSc)

## Abstract

Background: Systemic scleroderma (SSc) has multiple orofacial effects. The aim of this study was to analyze the expression of inflammatory mediators in biofilm samples. It was hypothesized that different expression levels and clinical associations might be drawn. Methods: A total of 39 biofilm samples from group 1 = SSc and group 2 = healthy control were examined for the expression levels of interleukin (IL)-2,-6, and -10; matrix metalloprotease (MMP)-9; and surface antigens CD90 and CD34 by quantitative real-time PCR and clinical parameters. Relative quantitative (RQ) gene expression was determined using the ∆∆CT method. Results: The mean bleeding on probing values (*p* = 0.006), clinical attachment loss (CAL) (*p* = 0.009), gingival recession (*p* = 0.020), limited mouth opening (*p* = 0.001) and cervical tooth defects (*p* = 0.011) were significantly higher in group 1. RQ expressions of IL-2 and CD34 were significantly lower, IL-6, MMP-9, and CD90 were significantly higher. There was a significant positive correlation of IL-6/MMP-9 and negative correlation of mouth opening/CAL and IL-6/CAL. Conclusion: Different expression levels of IL-2, IL-6, MMP-9, CD34 and CD90 were detected in biofilm samples from patients with SSc compared to control. An immunological correlation to the clinical parameters of mouth opening and CAL was shown; thus, we conclude that SSc might have an impact on periodontal tissues.

## 1. Introduction

Systemic scleroderma (SSc) is a complex chronic inflammatory autoimmune multisystem disease characterized by vasculopathy and fibrosis of the skin and internal organs [1]. It has been reported that 1 in 10,000 people are affected by SSc worldwide, with a prevalence between 38 and 341 total cases/million individuals [2,3]. The frequency peaks are between 45 and 64 years of age and three times more frequent in females [4]. From the time of diagnosis, the cumulative survival rate is 75% at 5 years, with SSc continuing to have the highest mortality compared to other rheumatic diseases, with heart failure or lethal cardiac arrythmias being the most common causes of death [1,5,6]. The clinical manifestations of SSc mainly include skin tightness, puffy fingers, sclerodactyly and digital open ulcers [1]. The extension and degree of SSc is evaluated using the modified Rodnan Skin Score (mRSS), and the definitive diagnosis will require the fulfilment of the criteria according to the 2013 European League Against Rheumatism (EULAR) and American College of Rheumatology (ACR) [7]. SSc is predominantly divided into the cutaneous limited (lcSSc) and diffuse subtypes (dcSSc). The limited type occurs in 43% of cases, and the diffuse type occurs in approximately 30% [3].

Even if the detailed mechanism and etiology remain unclear, it is known from the literature that the clinical and pathological manifestations are based on three different processes. First, autoantibodies and cell-mediated autoimmunity result from a dysfunctional immune system. Second, a disturbed proliferation of fibroblasts and thereby impaired perfusion of small vessels. Third, collagen deposition and other matrix components are increased in the skin and internal organs [3]. Ultimately, we assume that cytokines, such as transforming growth factor-beta (TGF-ß1), IL-6 and tumor necrosis factor-alpha (TNF-α), may be involved and consequently fibroblast differentiation and that the stimulation of extracellular matrix (ECM) is activated as an inflammatory response [8,9]. Moreover, different Th1 cell-associated cytokines or immunomodulatory mediators have been found in patients with SSc. Increased expression of the proinflammatory IL-2 and IL-6 has been detected in cell cultures derived from peripheral mononuclear cells and serum of patients with SSc [10,11,12,13]. The concentration of anti-inflammatory IL-10 was increased among patients with SSc in in vitro cultures with non-stimulated peripheral blood mononuclear cells [14].

Furthermore, increased serum levels of MMP-9 have been shown to be present in patients with SSc. Here, a connection between the increased concentration and the pathological change of the ECM is suspected [15].

Periodontitis (P) is a dysbiotic inflammatory disease with bacteria causing dysregulation of the immune response and leading to an excessive inflammatory reaction, periodontitis [14].

Some studies have observed a clinical correlation between SSc and P parameters. In a systematic review, six studies with a total of 84–178 patients were included. They reported a statistically significant association between SSc and oropharyngeal manifestations, including microstomia, limited mouth opening, decayed missing filled teeth (DMFT), clinical attachment loss (CAL), pocket depth (PD), less saliva and xerostomia, as well as reduced quality of life (QoL) [16].

However, we currently do not know whether the increase in periodontal parameters correlates with SSc on an immunological level or is associated in a bidirectional way. Only a few studies have investigated the potential reactions of immunomodulatory mediators in the gingiva, respective to gingival crevicular fluid (GCF) [8,17]. Gingiva tissue and periodontal ligament (PDL) samples were evaluated immunohistochemically and showed reduced TGF-ß1 levels in 30 patients with SSc and 30 patients with chronic P compared to 30 healthy controls [8]. Another study revealed higher TNF-α levels in the GCF of 20 patients with SSc as compared to that in 20 healthy controls [17]. The GCF represents an inflammatory mixture of multiple components (leukocytes, oral bacteria, periodontal cells) [18]. The collection can be done via paper strips, micropipettes or via the so-called crevicular washing [19]. Oral biofilm includes supra- or sub-gingival plaque components as well as the presence of periodontal tissue and can be sampled in the sulcus with removing it on the tooth surface [20].

However, the expression of inflammatory markers in oral biofilms or a correlation to different clinical parameters in patients with SSc remains unclear. Hence, we hypothesize that patients with SSc have different expression levels of inflammatory markers in the oral biofilm compared to healthy individuals. Thus, in this study, the expression of different immunomodulatory mediators in biofilm samples of patients with SSc and clinical parameters were examined and compared to those in healthy controls.

## 2. Materials and Methods

### 2.1. Study Design and Setup

The present study reports an observational prospective cohort trial at the Department of Oral and Maxillofacial Surgery, University of Erlangen-Nuremberg, from March 2019 to December 2020. The patients included in this study presented with SSc (group 1) or patients randomly seeking for dental treatment as healthy controls (group 2). The diagnosis of SSc and survey of the disease status were made by the Dermatology Clinic Department of the University Hospital Erlangen from clinical, serological and immunological results. Ethical approval (No.: 399_18 B) according to the Declaration of Helsinki for this study was obtained from the Ethics Committee of the Medical Faculties of the Friedrich-Alexander Universität Erlangen-Nürnberg, Germany and was also registered in the German clinical trial registry (DRKS00022956). In the same approved ethics application and registered clinical trial with the abovementioned numbers, patients with other underlying immunological diseases and equivalent examination protocols (inflammatory bowel disease and multiple sclerosis) were examined as disease groups as part of another study from the authors [18]. Concerning the healthy group which was equally formed of random patients seeking for dental treatment in this study, some patients may overlap.

Patients of both sexes were included if they were older than 18 years, willing to participate and had signed the informed consent. The included patients were assigned to group 1 or group 2 and were examined within one visit of an hour. Patients from both groups were matched by sex and age ±2 years.

The exclusion criteria were as follows:

- Smoking;
- Diabetes type I and II;
- Already diagnosed periodontitis or already undergone periodontitis treatment;
- Poor general health that does not permit a detailed dental examination;
- Toothlessness;
- Prior antibiotic treatment (neither systemic nor local administration for at least 4 weeks).

### 2.2. Data Extraction and Examination

The clinical, respective laboratory examination was based on the equivalent protocol and established in the laboratory of the Department of Oral and Maxillofacial Surgery to analyze biofilm samples with regard to inflammatory markers [21].

### 2.3. Clinical Examination

One calibrated examiner (A.L.) took part in the sampling procedures and assessed the clinical parameters.

The following parameters were collected:

- Bleeding on probing (BOP) in %
- Mombelli Plaque Index (mPI) graded from 0–3 (grade 0: no plaque detected by inspection and probing; grade 1: accumulation of plaque visible only by probing in the sulcus with a probe but not by eye; grade 2: visible plaque accumulation; grade 3: massive plaque accumulation)
- CAL in mm/% (distance from the cementoenamel junction to the bottom of the pocket/sulcus)
- PD -pocket depth at 6 sites using a PCP-12 probe (Hu-Friedy, Chicago, IL, USA) in %
- DMFT index
- Gingival recessions in mm/%
- Mouth opening in mm (maximum direction between the incisors of the lower and upper jaw); microstomia was defined at a value < 40 mm [22]
- Number of cervical tooth defects

Other parameters, such as the evaluation of panoramic X-rays (if available) regarding the temporomandibular joint, widening of the PDL, external cervical resorptions or other abnormalities, were also analyzed.

Two biofilm samples per patients were taken in the two deepest pocket sites by using a sterile curette and cotton rolls to isolate the saliva contamination. With the use of liquid nitrogen the samples could be transferred to the freezer at −80 °C. 

Moreover, the SSc disease score mRSS and medication were recorded.

The modified Rodnan Skin Score [23]:

- 0 = normal skin; no folds; no fibrosis
- 1 = mild fibrosis of the skin; fold is formed easily, fine folds are accepted
- 2 = moderate fibrosis of the skin; no fine folds
- 3 = severe fibrosis of the skin; no possibility to form folds

To collect the mRSS, 17 body regions are scored 0–3. The classification of 1–14 points indicates mild severity 1; 15–29 points indicates severity 2, which is equivalent to moderate severity; and 30–39 points indicates severity 3, which is equivalent to severe skin thickening/fibrosis. The final stage is reached at >40 points.

### 2.4. Examination of the Parameters in the Laboratory

With the use of reverse transcription quantitative polymerase chain reaction (RT-qPCR) the determination of cytokines and inflammatory mediators (IL-2, IL-6, IL-10, MMP-9, CD90 and CD34) could be detected in the biofilm samples as the total RNA was isolated.

The positive control was indicated by integrin alpha L, also called CD11a (ITGAL), to demonstrate that inflammatory material was present in the biofilm samples.

The ∆∆CT method was used to normalize the CT values using GAPDH as an endogenous control. The resulting ∆CT values were used for statistical analysis. A sample from the spleen was used as a positive control in all PCR investigations.

Higher ∆CT values indicate lower expression of the investigated target genes.

To show the relative change in expression rates, the fold change or relative quantification (RQ, (RQ, ∆∆CT-Method) was used.

To ensure the comparability of the parameters, they were taken in a standardized protocol.

To minimize the possible bias of sampling from the sulcus, only one practitioner (A.L.) took the samples. The term expression is used consecutively and describes the level of concentration of the mediators in the biofilm.

The primary outcome of the study was the different expression levels of the inflammatory markers in each group. Secondary outcomes were the assessment of clinical parameters (BOP, mPI, CAL, PD, DMFT, gingival recessions, maximum mouth opening, and cervical tooth defects).

### 2.5. Statistical Analysis

No study size was statistically determined in advance, as we first wanted to establish the methodology. Statistical analysis of the collected data was performed using SPSS, Version 25 software (IBM, Armonk, NY, USA). The Shapiro-Wilk test was used to test for normal distribution, which is based on no significance (*p* > 0.05) for the variable. For data analysis of non-parametric distributed variables, the Mann–Whitney U test (nonparametric test) was applied. In addition, variables in each group were tested for correlations using the Spearman test. A significance value of *p* ≤ 0.05 was considered statistically significant. If not stated otherwise, data are presented as the average ± standard deviation.

The analysis was performed by using the ∆Ct values (created from duplices) and the ∆∆Ct values were calculated. Duplicate values were used to determine the mean values of the Ct values of each sample and its associated endogenous control. The ∆Ct value is calculated as:

∆Ct (sample) = average Ct (target gene) − average Ct (GAPDH).

Based on all ∆Ct values within a group, an average ∆Ct value (average ∆Ct) can be calculated for the control group as well as the patient group:

Average ∆Ct (group) = sum of all ∆Ct/number of samples in a group.

The relative quantification (RQ) or the fold change (FC) was calculated according to:

∆∆Ct (target gene vs. control) = average ∆Ct (target gene) − average ∆Ct (control) and

RQ = 2 − ∆∆CT. An RQ value higher than 2 and less than 0.5 was considered significant. This indicates the change of an expression (x-fold) of the respective sample to the matched control sample (RQ-values).

## 3. Results

### 3.1. Study Population

A total of 17 patients with SSc (group 1), consisting of 12 females and 5 males, were included in this study. The mean age of these patients was 62.2 (SD = 10) years. The evaluated disease parameters, assignment to the subtype and disease activity (mRSS) are shown in Table 1. Group 1 had xerostomia in 55% of cases and sclerodactyly in 58% of cases. Moreover, 4 cases showed dull and dry mucosa, and 11 cases showed rhagades of the mouth.

The begin of the disease was the year of the initial diagnosis ranging from 1998 to 2019. In group 1, 94% of patients regularly took their medication, and 15 patients were treated with different immunosuppressants (mycophenolate mofetil (*n* = 4), iloprost (*n* = 7), tocilizumab (*n* = 1), rituximab (*n* = 1) and prednisolone (*n* = 2)), with no significant correlation between the clinical or laboratory parameters. Additional medications were antihypertensives (calcium antagonists (*n* = 7), angiotensin-1 antagonists (*n* = 1), angiotensin converting enzyme inhibitors (*n* = 2), loop diuretics (*n* = 1), and β-1 adrenoceptor antagonists (*n* = 1)) and proton pump inhibitors (*n* = 8), as well as thyroid hormones (L-thyroxine (*n* = 5)) and inhaled glucocorticoids (*n* = 2), with no significant correlation between the clinical or laboratory parameters.

The healthy group (group 2, *n* = 22) consisted of 15 females and 7 males with a mean age of 61.5 (SD = 9) years.

### 3.2. Clinical Parameters

#### 3.2.1. Bleeding on Probing (BOP) and Mombelli Plaque Index (mPI)

The mean values for BOP were significantly higher in group 1 as compared to those in group 2 (*p* = 0.006, Table 2). However, the mPI was not significantly different between the two groups (*p* = 0.451, Table 2), with patients with SSc showing increased values.

#### 3.2.2. Clinical Attachment Loss (CAL)

The mean values (% and mm) of CAL did significantly differ between groups 1 and 2 (*p* = 0.035 and *p* = 0.009), as shown in Table 2. The patients in group 1 had a twofold higher CAL as compared to that in group 2.

#### 3.2.3. DMFT Index and PD

Regarding the DMFT a higher value was seen in group 1 but without any significance. However, group 1 showed a higher amount of PD ≥ 4 mm in 14.83% of the teeth compared to the group 2 (11.58%).

#### 3.2.4. Gingival Recession

Group 1 had significantly higher values (% and mm) of gingival recession when compared to group 2 (*p* = 0.020). Group 1 had twofold more gingival recessions than group 2. Moreover, the length of this recession was higher compared to controls (group 2), with statistical significance (*p* = 0.020) (Table 2).

#### 3.2.5. Mouth Opening

The mean value of the maximum mouth opening distance differed significantly between groups 1 and 2 (*p* = 0.001), with an average opening distance of 10 mm in group 1. 47% of group 1 had a mouth opening distance < 38 mm.

#### 3.2.6. Cervical Tooth Defects

The mean values of cervical tooth defects differed significantly (*p* = 0.011) between group 1 (*n* = 5.82) and group 2 (*n* = 2.36), as shown in Table 2.

#### 3.2.7. Cervical Extern Resorptions/Radiological Findings

In *n* = 17 panoramic x-rays within group 1 no cervical resorptions on teeth could be found. However, changes of the temporomandibular joint, in terms of arthrotic symptoms (*n* = 3), could be found. Moreover, widening of the PDL involving *n* = 62 teeth (*n* = 12 anterior teeth and *n* = 50 posterior teeth) was present within group 1.

### 3.3. Cytokine Expressions

The expression of the included cytokines and immunomodulatory expression did not differ significantly between groups 1 and 2, as shown in Table 2. However, if the mean values are considered descriptively, at least tendencies emerge. IL-6 and IL-10, CD90 and CD11a were overexpressed in group 1, whereas CD34 had lower expression levels in group 1. The expression of MMP-9 was the highest of all parameters considered overall and had a higher mean value in group 1. Relative quantification (RQ) was further used to evaluate expression levels more precisely. Thus, a significant lower expression of IL-2 and CD34 and a significant overexpression of IL-6, MMP-9, CD90 and CD11a were observed in group 1 (Table 2).

#### Correlation between Clinical and/or Laboratory Parameters

**Figure 1:**
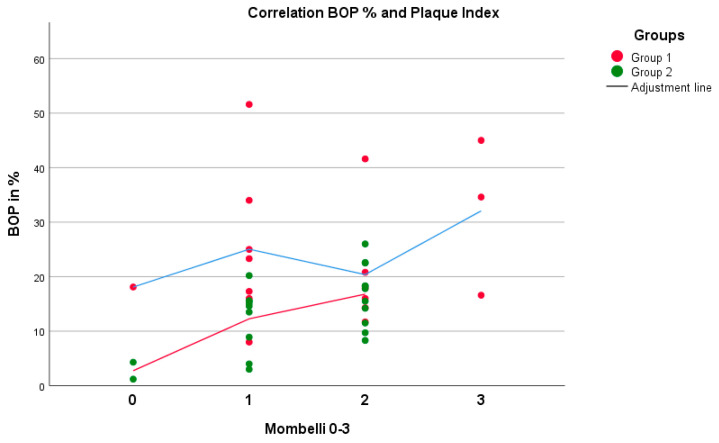
There was a positive correlation (higher values of BOP indicate higher values of mPI) between the parameters BOP and mPI, with no significant difference in group 1 (*p* = 0.809, rS = 0.063) but with a significant difference in group 2 (*p* = 0.014, rS = 0.514) (Spearman test).

**Figure 2:**
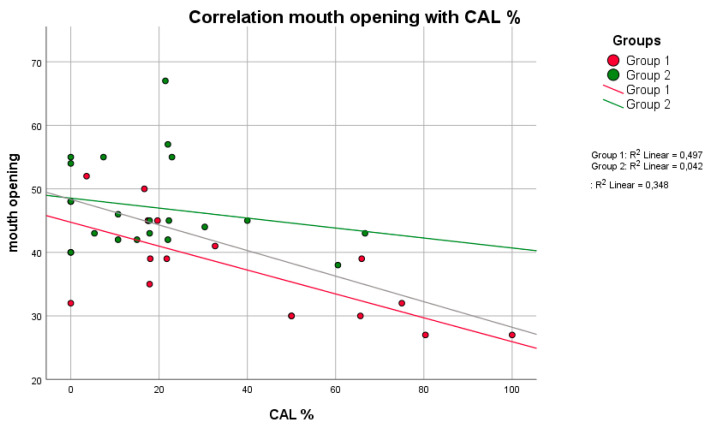
There was a negative correlation (higher mouth opening indicates lower values of CAL and vice versa) between the parameters mouth opening and CAL in both groups, with a significant difference in group 1 (*p* = 0.011, rS = −0.722) (Spearman test).

**Figure 3:**
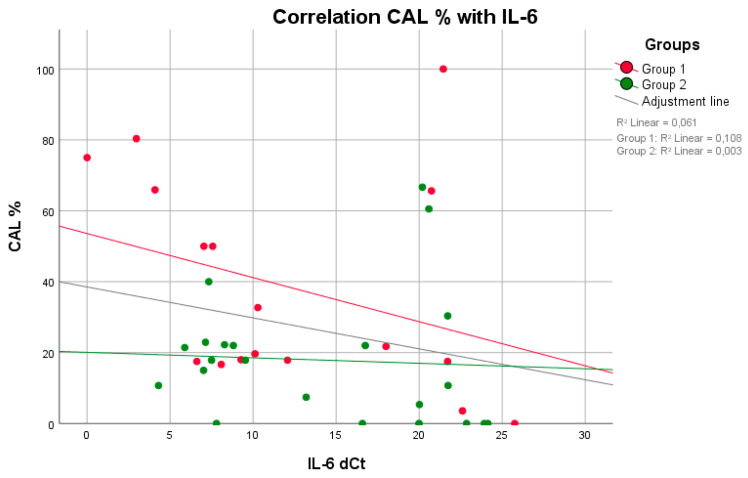
A negative correlation (higher values of IL-6 indicate lower values of CAL and vice versa) was observed between the parameters IL-6 and CAL in both groups, with a significant difference in group 1 (*p* = 0.049, rS = −0.483) (Spearman test).

**Figure 4:**
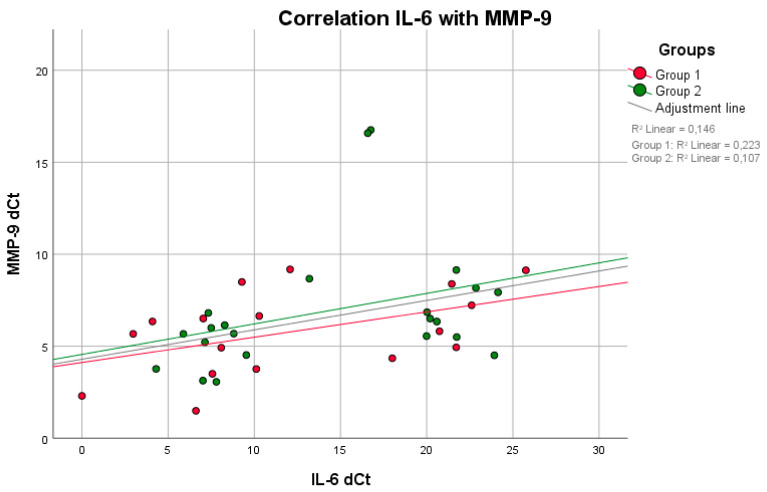
There was a positive correlation between IL-6 and MMP-9 (higher IL-6 indicates higher MMP-9 values) in both groups, with a significant difference in group 1 (*p* = 0.034, rS = 0.517) (Spearman test).

**Figure 5:**
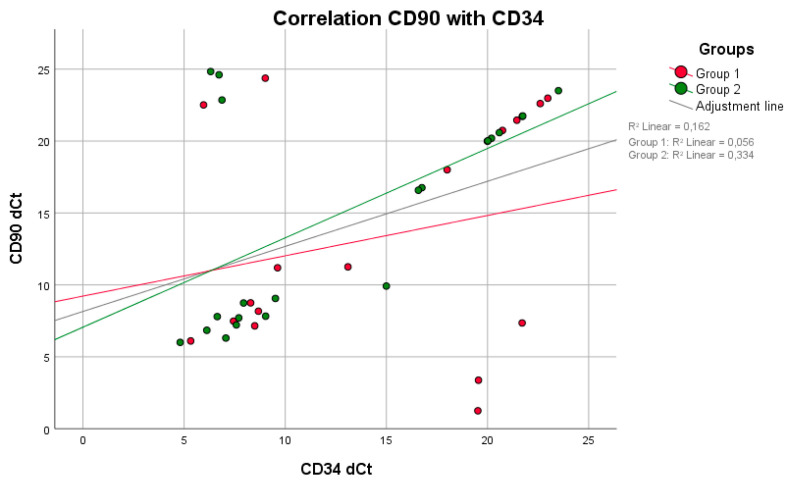
There was neither a positive nor a negative correlation between the parameters CD90 and CD34 in either group (Spearman test).

No positive correlation between mPI values and BOP (*p* = 0.809, rS = 0.063) was observed in group 1. Higher BOP values were not associated with higher mPI values in group 1 (Figure 1). However, these parameters were equivalent to those in group 2 (*p* = 0.014, rS = 0.514). A negative correlation between the maximum mouth opening and CAL (*p* = 0.011, rS = −0.722) was seen in group 1 (Figure 2). With reduced mouth opening values, higher CAL values occurred and vice versa. The same applied for PD (*p* = 0.015, rS = −0.581). No negative correlations of these parameters were seen in group 2. A negative correlation between CAL and IL-6 was shown in group 1 when compared to group 2 (Figure 3). In patients with higher CAL values, the expression of IL-6 was lower or vice versa in group 1 (*p* = 0.049, rS = −0.483). A positive correlation between IL-6 and MMP-9 (*p* = 0.034, rS = 0.517) in group 1 (Figure 4) was shown in terms of higher IL-6 indicating higher MMP-9 values. No positive or negative correlation was seen between CD90 and CD34 in group 1 (*p* = 0.264, rS = 0.287) (Figure 5). No further correlations could be drawn regarding the mRSS and/or other parameters.

## 4. Discussion

Periodontitis develops as an immune response to an imbalance of oral bacteria that prevails over a long period of time, which can also be influenced by many environmental factors, such as smoking or diabetes, and is characterized by the degradation of periodontal tissue and bone [24,25]. Due to the immunologically controlled response, many different modulators (e.g., cytokines, MMP, etc.) play a role in this process. Basically, it is known that TNF-α, IL-6 or IL-1 are released and attract further B- and T-cell controlled cells, thereby initiating destruction [14]. Although the exact mechanism of the development of SSc remains unclear, it is known that it also develops based on an immune response and affects the vascular system, internal organs and skin with excessive fibrosis and degradation of connective tissue [1,3]. Due to fibrosis of the skin, many orofacial changes also result, especially restricted mouth opening [16]. Coupled with limited movement of the fingers and hands and sclerodactyly, patients with SSc suffer from limited oral hygiene ability [26]. Studies have also observed decreased QoL in this context [1,16]. In addition, a recent study showed that patients with dcSSc (*n* = 28) in particular had a decreased salivary flow rate with reduced pH values, which correlated positively with DMFT and mRSS [27]. It is unclear whether the increase in DMFT and decreased salivary flow rate are due to SSc itself or due to Sjögren’s syndrome concomitant with SSc.

We also found increased periodontal parameters in the SSc group as compared to those in the healthy group, which corresponds to the results from the current literature [8,17,28,29]. In particular, BOP, CAL, numbers of gingival recessions and cervical defects, and limited mouth opening were significantly increased.

Therefore, are the increased periodontal parameters in SSc patients due to a reduced salivary flow rate, restricted mouth opening, lack of dexterity and associated restricted oral hygiene, or are they also due to immunological processes caused by SSc itself?

Clinical studies have already provided evidence of immunological modulators in the serum of patients with SSc, which are also known in the context of periodontitis. Thus, proinflammatory IL-2 is thought to be important in both diseases. In 448 sulcus fluid samples, IL-2 was detected in infected pockets in patients with P [30]. In patients with SSc, IL-2 has been previously shown to be decreased in serum [10,31]. This observation also fits with our results, in which patients with SSc, although not in serum but in biofilms, showed significant lower expression of IL-2 when compared to healthy individuals. Decreased expression of proinflammatory markers was also reported in smokers, as confirmed by a study in which smokers and non-smokers were compared in terms of cytokine expression in plaque samples. Smokers showed significantly reduced TNF, respective pro-inflammatory cytokine levels when compared with nonsmokers [32]. However, we excluded smokers to eliminate this confounding effect, yet patients with SSc showed significantly more PD and CAL than healthy individuals.

Furthermore, studies also found elevated levels of proinflammatory IL-6 in the serum of patients with dcSSc [11,12]. Additionally, increased concentrations of IL-6 could be confirmed in the saliva of patients with P [33]. This was increased when compared with healthy subjects, particularly during the initiation response, but remained at the original level during further progression of P [34]. In our patient population, although only 6 patients with dcSSc were included, we also observed a significant overexpression of IL-6 in the biofilms of patients with SSc compared to healthy individuals. In addition, IL-6 correlated strongly and negatively with CAL, so we can also assume that IL-6 indicates a current inflammatory event initially without unconditional clinical impact. Thus, blocking such receptors not only prevents the extent of SSc itself but also might show effects on the periodontium [11,35].

Metalloproteases are known to be major contributors to ECM degradation and are therefore of great importance in SSc. Significantly decreased serum concentrations of MMP-9 in patients with SSc and a negative correlation of MMP-9 with the mRSS were demonstrated [36,37,38]. In P, however, the expression of MMP-9 was increased in saliva and sulcus fluid when compared to healthy individuals [34,39,40,41]. Within this study, patients with SSc showed a significant overexpression of MMP-9. Moreover, the expression of MMP-9 correlated strongly and positively with that of IL-6 in the SSc group, so we can assume an acute inflammatory status of the periodontium. This observation would also explain, in particular, the significantly increased BOP without concomitantly increased mPI in patients with SSc. BOP is mainly associated with initial inflammation [34]. Furthermore, this result shows that limited oral hygiene alone cannot be a problem. This also correlates partially to the following study. In contrast, they observed an increased plaque index (PI) in patients with SSc, which also correlated negatively with CAL, so the authors considered poor oral hygiene to be the cause only to a limited extent [28]. The fact that this positive correlation can also be observed in healthy individuals is because we also had to take samples from the deepest parts of the sulcus in healthy individuals, thus enabling an inflammatory reaction in the early stadium. Thus, BOP also correlates positively with the mPI in healthy individuals.

Oral hygiene or restricted mouth opening leading to increased parameters in patients with SSc is also shown by further observations. A case-control study at least could contradict this by finding no association between mRSS and oral hygiene [42]. Our patients showed at least a significant positive correlation between decreased mouth opening and CAL. However, no correlation with mRSS could be drawn, whereas another study showed a positive correlation between CAL and mRSS in patients with SSc [26].

The literature indicates that the surface antigens CD90 and CD34 correlate and are associated with increasing fibrosis of the skin and mRSS [43,44,45]. In particular, CD90 cells seem to replace CD34 cells and show a negative correlation with skin fibrosis [43,44]. Within these patients suffering from SSc, we were able to demonstrate this negative correlation when CD90 was overexpressed and CD34 was simultaneously under-expressed compared with healthy individuals in the biofilm samples. However, they did not show a significant negative correlation. Additionally, no correlation with mRSS could be shown, but the number of patients with SSc was very low. Immunohistochemically, neither surface antigen has been detected in either healthy or patients with P thus far [46]. This argues for the methodology chosen here for further studies and the inclusion of patients with P.

The anti-inflammatory IL-10 showed no significance in the SSc group compared with the healthy group. Although this cytokine in the serum of patients with SSc also provided conclusions about the degree of fibrosis and mRSS [47], the data situation, in this respect, has been very limited to date. With regard to IL-10, no conclusions can be drawn within this study.

To our knowledge, there has been only one case report describing increased cervical resorptions in patients with SSc [48]. None of our included patients showed this phenomenon, although significantly more gingival recessions and cervical tooth defects were observed compared to healthy individuals, which also correlated positively with restricted mouth opening and CAL. Moreover, radiological findings, in terms of widening of the PDL or changes in the temporomandibular joint (TMJ), could be found in these patients, according to the literature describing these changes [22,49].

There are shortcomings and limitations in this study that need to be mentioned and critically discussed. First, the small number of patients included in this study has to be mentioned because only a few patients with SSc patients were available. However, this small number of patients certainly reduced the evidence for the presence of differential expression among groups.

Moreover, the markers considered do not indicate the severity of periodontal changes. On the one hand, because they are non-specific, also because of the methodology chosen here and, on the other hand, because there were no temporally separated examination time points. This should be considered for follow-up studies.

Since the literature shows that especially in healthy patients little GCF [19] can be collected and we want to establish a different type of methodology to still be able to analyze mRNA, we decided against collecting GCF. Even though the results may be reduced because it is not clear to what extent the GCF correlates with the biofilm, the simplified col-lection with the curette shows that levels of modulators can be detected.

Furthermore, this study did not determine the bacterial composition or include patients with periodontal disease, which was why no link between SSc and periodontitis or differences in its severity could be proven by these study results, and further studies are necessary on this topic.

## 5. Conclusions

Patients showed significantly increased periodontal parameters (CAL and BOP), increased gingival recession, cervical defects and decreased mouth opening. However, they also showed increased concentrations of MMP-9, IL-6 and CD90 in the biofilm samples, which partially correlate with and account for the increased periodontal parameters. Further studies are needed to analyze inflammatory marker expression in patients with SSc and periodontitis to find similarities between these diseases and to provide an explanation for the clinical observation of the prevalence of periodontitis in patients with SSc.

## Figures and Tables

**Table 1:** The symptomatology of SSc diseases and the number of patients (*n*) in the subdivided groups of SSc (lcSSc and dcSSc).

Clinical Parameter	lcSSc (*n* = 11)	dcSSc (*n* = 6)
Raynaud Syndrome	10	6
Sicca Symptomatic	6	5
Xerostomia	3	3
Esophageal motility disorder	8	6
Restricted lung function	5	5
Digital ulcers	2	3
Sklerodaktyy	7	3
mRSS (Ø)	4.9	11.6

**Table 2:** The ∆CT values of cytokines (IL-2, IL-6, and IL-10), MMP-9, CD90 and CD34 (CD11a as positive control) and the mean values of clinical parameters (BOP, mPI, CAL, PD, DMFT, number of teeth, gingival recession, maximum mouth opening and cervical tooth defects) in group 1 (SSc) and group 2 (healthy control) with the corresponding *p*-value (significance level *p* < 0.05) and RQ values (fold change) and significance at RQ value > 2 for overexpression and RQ value < 0.5 for lower expression are shown. Significances were marked as bold * in BOP, CAL, gingival recession, maximum mouth opening and cervical tooth defects. Significant RQ values were marked as bold ^#^ of IL-2, IL-6, MMP-9, CD90 and CD34 and the positive control CD11a (Mann–Whitney-U-test).

	Group 1 (*n* = 17) (SD)	Group 2 (*n* = 22) (SD)	*p*-Value	RQ
CT IL-2	23.00 (2.43)	21.98 (4.76)	0.679	**0.49** ^#^
CT IL-6	13.02 (7.42)	14.33 (6.99)	0.479	**2.48** ^#^
CT IL-10	10.81 (7.22)	11.29 (6.11)	0.590	1.40
CT MMP9	5.80 (2.28)	6.93 (3.54)	0.590	**2.19** ^#^
CT CD90	13.22 (7.86)	15.03 (7.13)	0.552	**3.52** ^#^
CT CD34	14.26 (6.66)	12.83 (6.62)	0.396	**0.37** ^#^
CT CD11a	6.11 (4.34)	9.12 (6.68)	0.193	**8.07** ^#^
BOP (in %)	24.22 (12.59)	13.66 (6.72)	0.006 *	-
mPI (grade 0–3)	1.65 (0.86)	1.41 (0.66)	0.451	
CAL (%)	38.35 (29.78)	17.86 (18.55)	0.035 *	-
CAL Ø (mm)	1.95 (1.73)	0.76 (0.95)	0.009 *	-
PD ≥ 4 mm (%)	14.83 (9.24)	11.58 (8.99)	0.178	-
DMFT	20.53 (5.23)	18.00 (6.36)	0.150	-
Gingival recession (%)	38.14 (29.71)	16.90 (18.11)	0.020 *	-
Gingival recession Ø (mm)	1.14 (1.10)	0.43 (0.56)	0.020 *	-
Maximum mouth opening (mm)	37.53 (7.95)	47.14 (7.12)	0.001 *	-
Cervical tooth defects (*n*)	5.82 (5.11)	2.36 (2.64)	0.011 *	-

## Data Availability

The datasets used and/or analyzed during the current study are available from the corresponding author on reasonable request.
